# A rare diagnosis of Langerhans cell histiocytosis made on thyroid histology with coexisting papillary thyroid cancer and AVP deficiency

**DOI:** 10.1530/EDM-23-0050

**Published:** 2024-04-22

**Authors:** R K Dharmaputra, C M Piesse, S Chaubey, A K Sinha, H C Chiam

**Affiliations:** 1 Cairns and Hinterland Hospital and Health Service, Cairns, Queensland, Australia; 2 Department of Endocrinolgy and Diabetes, Cairns Hospital, Cairns, Queensland, Australia; 3 Cairns Diabetes Centre, Cairns, Queensland, Australia; 4 Gold Coast Hospital and Health Service, Gold Coast, Cairns, Queensland, Australia; 5 Department of Surgery, Cairns Hospital, Cairns, Queensland, Australia

**Keywords:** Adult, Male, Asian - Filipino, Australia, Bone, Pituitary, Thyroid, Thyroid, Endocrine-related cancer, Unique/unexpected symptoms or presentations of a disease, April, 2024

## Abstract

**Summary:**

A 48-year-old Asian male, presented to the hospital for an elective total thyroidectomy in the context of 6.3 cm thyroid nodule. The fine needle aspiration cytology of the nodule confirmed papillary thyroid cancer (PTC) with some atypical histiocytes. He has a history of idiopathic arginine vasopressin deficiency (AVP-D) and has been taking oral DDAVP 100 µg daily, self-adjusting the dose based on thirst and polyuria. Additionally, he also has a history of recurrent spontaneous pneumothorax. His total thyroidectomy was aborted due to significant intraoperative bleeding, and his admission was further complicated by post-operative hyponatraemic seizure. Thyroid histology revealed the diagnosis of Langerhans cell histiocytosis (LCH), and further investigation with contrast CT demonstrated multi-organ involvement of the thyroid, lungs, and bones.

**Learning points:**

- Langerhans cell histiocytosis (LCH) is a condition that can affect one or more organ systems, including the pituitary, where it can present as AVP deficiency. Strict monitoring of fluid balance, as well as serial monitoring of serum sodium, is essential in all patients with AVP-D in the perioperative setting.
- Iatrogenic hyponatraemic seizure is an uncommon but serious complication of DDAVP treatment in hospitalised patients with AVP-D. DDAVP dosing must be carefully monitored.
- LCH with multisystem involvement is an important mimic for metastatic conditions, and histological diagnosis is essential to guide treatment and prognosis.
- Although LCH without bone marrow involvement is unlikely to increase the risk of bleeding, its effect on tissue integrity may make surgery more challenging.
- *BRAF-V600E* mutation is an important driver mutation and a potential therapeutic target in the treatment of LCH.

## Background

Langerhans cell histiocytosis, or LCH, is a haematological condition that results from the formation of granulomatous lesions, which are aggregates of langerin-positive (CD207+) histiocytes. It leads to the accumulation of inflammatory infiltrates in organs such as bones, skin, lungs, and the pituitary (1). Although more common in the paediatric population, LCH may also be encountered in adults. Abnormalities of the hypothalamic–pituitary axis have been observed in 68% of patients with positive intracranial imaging. AVP-D is the most common endocrinopathy encountered in LCH and has been reported in up to 50% of cases (2). Positive intracranial imaging may range from infundibular thickening of the pituitary to a partial or complete empty sella, or a pituitary infiltrative lesion (3).

The diagnosis of LCH can be challenging and can only be proven through tissue histology and immunohistochemistry. LCH has a heterogeneous clinical presentation, ranging from single-organ involvement to multisystemic disease, and may mimic other conditions such as metastatic malignancies, infections, IgG4 disease, and other more common granulomatous disorders. Some case reports have demonstrated the coexistence of LCH and papillary thyroid cancer (PTC) (4, 5); however, there is currently no known association between the two conditions. *BRAF* mutation is a common driver mutation found in adults with LCH and PTC. It is also a well-known treatment target in LCH with CNS involvement and in redifferentiation therapy for radioiodine-refractory PTC (6, 7). Synchronous *BRAF* mutation in LCH and PTC has previously been described (2).

## Case presentation

A 48-year-old Asian male, presented to the hospital for an elective total thyroidectomy in the context of a large thyroid nodule. A neck ultrasound was performed due to the concern of midline swelling, which demonstrated a 6.3 × 5.1 × 3.1 cm lesion on the isthmus of the thyroid, and cervical lymphadenopathy was noted. Fine needle aspiration (FNA) confirmed papillary thyroid cancer with atypical histiocytes. He was euthyroid preoperatively. NL has a history of idiopathic AVP-D diagnosed 20 years ago in Philippines and has been taking 100–200 µg oral DDAVP daily. The history of AVP-D was well documented, and all clinicians involved were aware of this diagnosis. His serum sodium in the community ranges between 128 and 142 mmol/L. His other medical conditions include vitiligo, hypertension, gastro-oesophageal reflux disease, recurrent pneumothorax, and an 8 mm left adrenal incidentaloma. He is an ex-smoker and consumes 3–5 glasses of red wine daily.

During the thyroidectomy, the standard technique of capsular dissection was carried out on the left thyroid lobe. Difficulties were encountered due to the significant vascularity and friability of the gland and its adherence to surrounding tissues. The gland appeared to be enlarged and inflamed, with a portion of the left sternothyroid muscle densely adherent to the thyroid gland. In dissecting off the fibres, the thyroid capsule was torn, resulting in significant bleeding. Various techniques were attempted to achieve hemostasis, including pressure, surgical clips, standard diathermy coagulation, thunderbeat energy device dissection/spot coagulation, suture-ligation, and haemostatic matrix agent, but only resulted in temporary haemostasis. Further thyroid dissection caused recurrent and persistent bleeding, which was difficult to control. Intraoperative surgical consultations and assistance from three other experienced surgeons were sought, and all of them found similar difficulties with the procedure. The persistent bleed settled slowly after the superior and inferior thyroid vessels were ligated, and the thyroid isthmus was ligated and cut. The left thyroid lobe and ipsilateral cervical lymph nodes were removed in segments. A consensus decision was made not to continue with the right thyroid lobectomy due to the significant blood loss and difficulties already encountered.

He was admitted to the intensive care unit (ICU) post-operatively for blood transfusion and hemodynamic control due to the significant intraoperative difficulty. He received 1 L of i.v. crystalloid and was noted to have a robust urine output of up to 300 mL/h. His serum sodium on the day of ICU discharge was 129 mmol/L, and i.v. fluid was discontinued.

**Figure 1:**
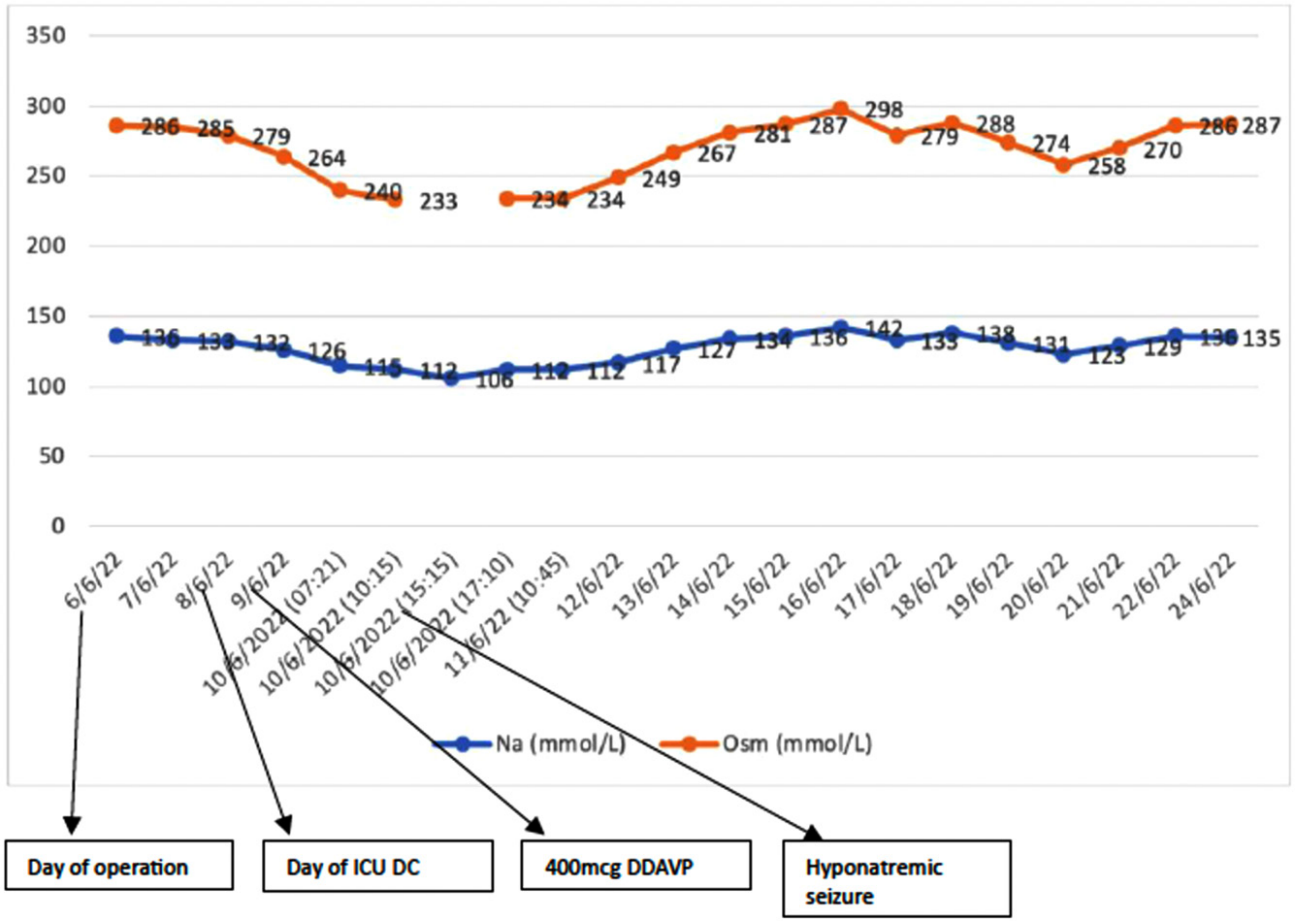
Serum sodium and osmolarity.

Figure 1 describes the trend of serum sodium and osmolarity throughout the hospital admission. Further details regarding fluid balance and DDAVP dose are provided in Table 1. We note that the fluid balance record in this case is more reflective of the 24-hour urine output as oral intake and i.v. fluid were not accurately measured and recorded. Following ICU discharge, the patient was monitored with daily blood tests. His fluid balance was not recorded in the electronic record. His sodium trended down, from 132 mmol/L on day 1 of discharge from ICU to 126 mmol/L on day 2, and 115 mmol/L on day 3 of ICU discharge. At this point, the precipitous drop was recognised, and endocrinology consultation was sought. Paired serum and urine osmolality and urine sodium demonstrated hypotonic hyponatraemia with a SIADH-like state, with a serum osmolality of 240 mmol/L, urine osmolality of 319 mmol/L, and an inappropriately elevated urine sodium excretion of 91 mmol/L. Strict fluid balance with hourly urine output measurement and 1 L fluid restriction was commenced. A 100 mL hypertonic saline bolus was administered due to gradually worsening confusion that ultimately culminated in hyponatraemic seizure and status epilepticus, with a nadir in serum sodium of 106 mmol/L. He was readmitted to the ICU for ongoing hypertonic saline infusion. At the time of his initial endocrine review, a bottle of 200 µg DDAVP tablets was found at his bedside, raising suspicion of iatrogenic hyponatraemia. This was later confirmed by the patient who admitted to self-administering two tablets of DDAVP in addition to his prescribed dose (24 h total of 500–600 µg) to self-treat his polyuria. Following initial sodium correction in ICU, oral DDAVP was recommenced under the guidance of endocrinology. His daily dose of DDAVP was further titrated to serum sodium and urine output to a final dose of 100 µg mane and 50 µg nocte.

**Table 1:** Serum sodium, osmolarity, and DDAVP dose.

Date and time	Na (mmol/L)	Osm (mmol/L)	Urine Osm (mmol/L)	Fluid balance (mL)	DDAVP (μg)
6 June 2022	136	286			100
7 June 2022	133	285		−345	100
8 June 2022	132	279			100
9 June 2022	126	264			400
10 June 2022					
07:21	115	240	319		100
15:15	112	233			
17:10	106				
11 June 2022					
10:45	112	234			
6 June 2022	112	234			100
6 June 2022	117	267			
14 June 2022	134	281		450	100
15 June 2022	136	287		−3655	100
16 June 2022	142	298		−1060	150
17 June 2022	133	279	221	−4020	150
18 June 2022	138	288		−735	200
19 June 2022	131	274		−1560	200
20 June 2022	123	258		−3745	150
21 June 2022	129	270		−2965	150
22 June 2022	136	286			150
24 June 2022	135	287			150

## Investigation

**Figure 2:**
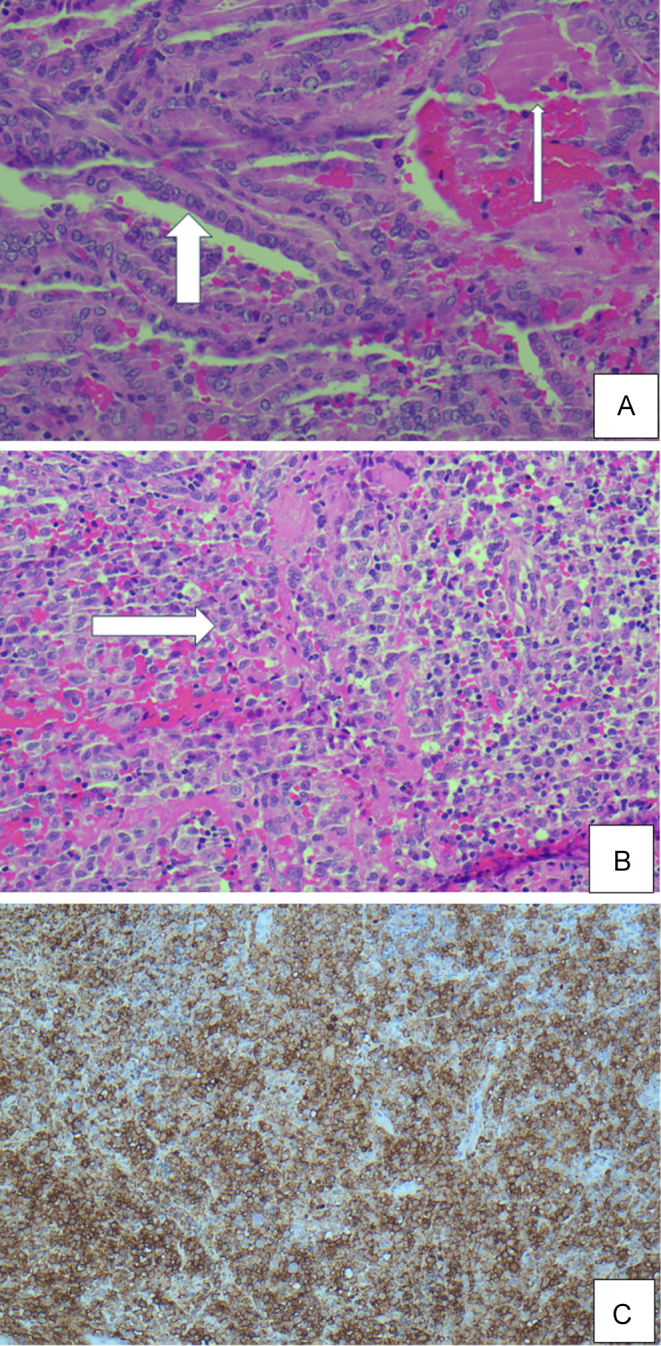
Thyroid histology slide demonstrating coexisting classical PTC and LCH. (A) Papillary thyroid cancer: enlarged, elongated pale nuclei with irregular membrane papillary structures lined by tumour cells with nuclear clearing and nuclear grooves. Thick colloid in right upper corner. (B) LCH on H&E stain: mass-forming collections of Langerhans histiocytes with clefted nuclei/coffee bean nuclei. Some eosinophils are seen. (C) LCH CD1a+ immunostaining: cells with reactive cytoplasm are stained brown.

Figure 2 outlines the histopathological findings from the thyroid tissue. Thyroid histology demonstrated a mixed pathology of classical PTC and LCH. In relation to the coexistence of LCH and PTC, BRAF mutation analysis of the PTC was negative, suggesting distinct pathological processes driving the development of LCH and PTC. Histiocytic invasions were observed in the skeletal muscles, thyroid follicles, and lymph nodes with marked sinus expansion. There was no evidence of metastatic papillary thyroid cancer in lymph nodes.

**Figure 3:**
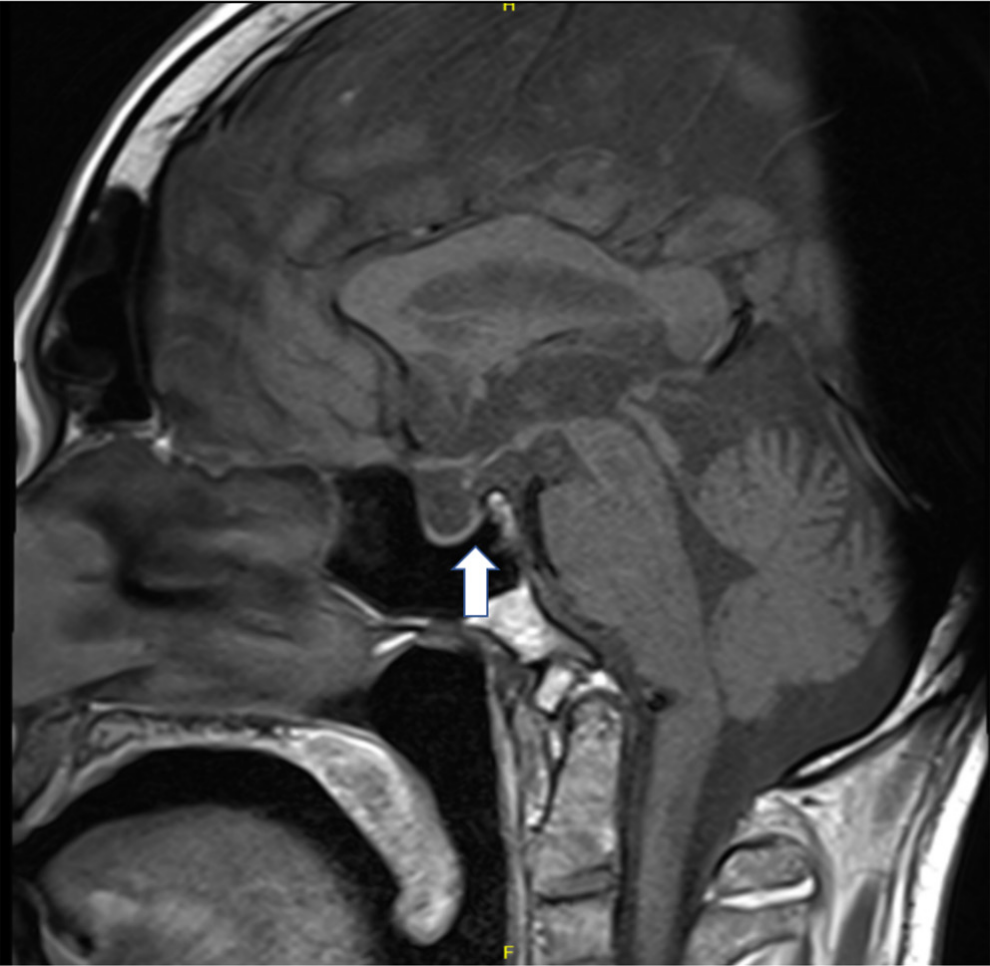
Sagittal T1-weighted MRI imaging with empty sella appearance, with absent posterior pituitary bright signal labelled with arrow.

**Figure 4:**
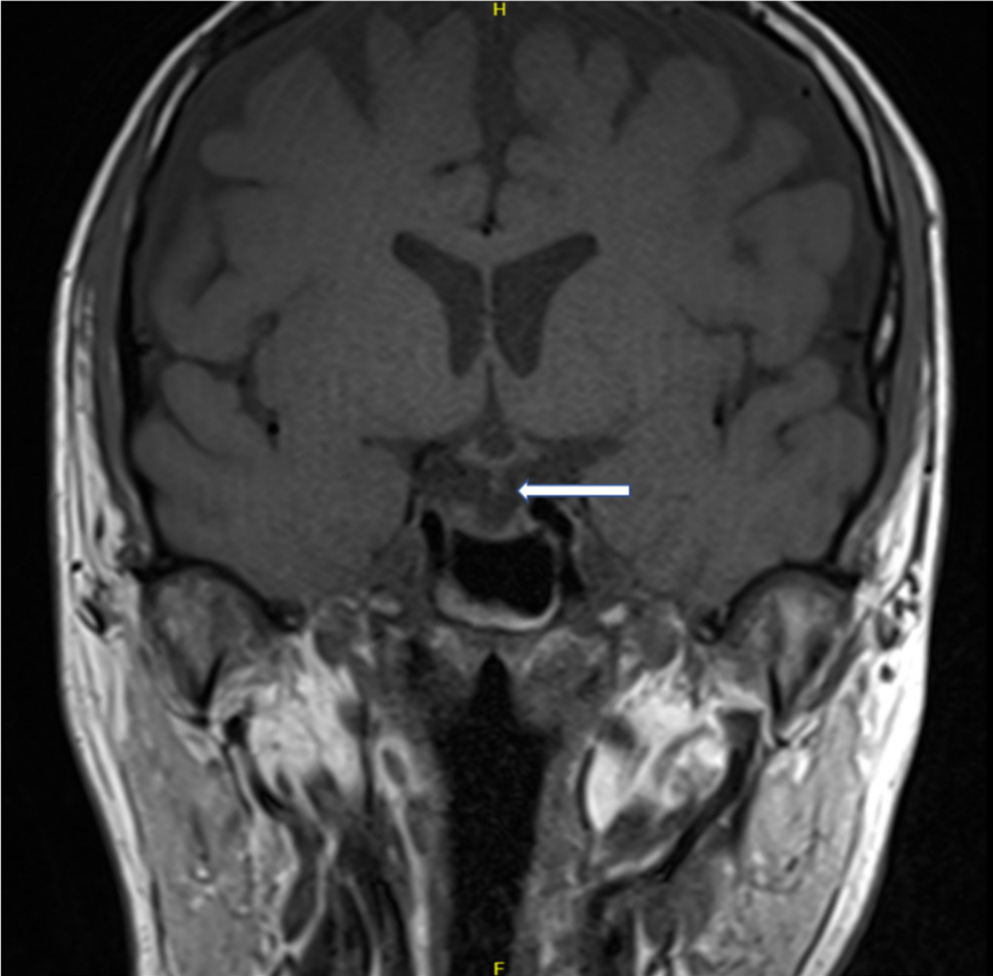
Coronal T1-weighted MRI imaging demonstrating the pituitary stalk which is normal in thickness, labelled with arrow.

MRI pituitary performed following the seizure demonstrates the appearance of an empty sella with absent posterior pituitary bright signal on T1, as outlined in Fig. 3. There are no radiological or clinical features suggestive of CNS involvement of LCH. Figure 4 demonstrates the normal appearance of the pituitary stalk without thickening, which can also be a feature of LCH or hypophysitis.

In view of the finding of an empty sella, a full anterior pituitary hormone assessment was performed (Table 2), which was unremarkable.

**Table 2:** Anterior pituitary hormones.

Test	Values	RR
Prolactin, mU/L	414	55–300
ACTH, ng/L	28	10–50
Cortisol, nmol/L	536	
TSH, mIU/L	3.17	0.55–4.78
T4, pmol/L	12.3	11.5–22.7
FSH, U/L	4.9	1.4–18
LH, U/L	5.9	1.5–9.3
GH, μg/L	0.17	0.05–3.0
IGF-1, nmol/L	20	9–29

ACTH, adrenocorticotrophic hormone; FSH, follicle-stimulating hormone; GH, growth hormone; IGF-1, insulin-like growth factor 1; LH, luteinizing hormone; RR, reference range; TSH, thyroid-stimulating hormone.

Following the thyroid histology and MRI pituitary findings, we conclude that multisystemic LCH is the most likely aetiology responsible for the pre-existing AVP-D. Further radiological investigations revealed other features of LCH, including multiple cystic lung lesions and multiple expansile radiolucent lesions on the right occipital condyle and the right lamina of L4 and L5 vertebrae. Fluorodeoxyglucose-positron emission tomography demonstrated increased uptake in the thyroid gland and focal uptake on the right 5th rib.

### Treatment, outcome, and follow-up

Following left hemithyroidectomy and the diagnosis of LCH, NL was discharged with endocrinology, general surgery, and haematology outpatient follow-up. His oral desmopressin dose was further titrated to a dose of 100 µg twice daily, and he was commenced on cytarabine-based chemotherapy with adjuvant external beam radiation therapy to the vertebral lesion.

## Discussion

LCH is a rare haematological condition with an incidence of one to two cases in 1 million adults (8). Patients with LCH are at high risk of developing central endocrinopathies, including AVP-D. Twenty-five percent of AVP-D cases were diagnosed at the same time as LCH itself; 4% of diagnoses were made prior to LCH; and 18% of cases were diagnosed concomitantly or after LCH (2). In relation to anterior pituitary hormones, the growth hormone and gonadotropin axes are most affected (9). Given the highly heterogenous manifestations, LCH is therefore an important diagnosis to consider in patients presenting with central endocrinopathy with the involvement of other organ systems. Thyroid involvement with extrathyroidal extension has previously been described and may contribute to increased surgical risk and complexity, as seen in our case (4, 5).

LCH involving thyroid tissue is extremely rare, and our case is unique as the diagnosis of LCH was made from thyroid histology. Although a case of LCH and PTC with synchronous BRAF mutation has been reported, our patient has a BRAF-negative PTC, suggesting a different driver mutation might be responsible for the two separate pathologies. Although LCH has been associated with an increased prevalence of solid organ and haematological cancers, the relationship between LCH and thyroid cancers remains unclear, with only a few publications reporting synchronous presentation of LCH and PTC.

Regarding histological findings, although atypical histiocytes were detected in the skeletal muscles, thyroid tissue, and lymph nodes; papillary thyroid cancer cells were confined to the thyroid without any evidence of lymphovascular invasion. This case was further discussed in a multidisciplinary setting involving endocrinologists, thyroid surgeons, radiologists, and anatomical pathologists, and a decision was made to forego completion of thyroidectomy due to the significant risk associated with repeat operation and the overall impression of low-grade papillary thyroid cancer with low risk of recurrence.

Although LCH without bone marrow involvement is unlikely to increase the risk of bleeding, it is evident that the effect of histiocytic infiltrate on tissue integrity, like other infiltrative disorders, can make any surgery more challenging, as seen in our case.

In addition to intraoperative challenges, this case also illustrates the importance of strict perioperative fluid and electrolyte monitoring in individuals with AVP-D. In addition to iatrogenic hyponatraemia, it is postulated that surgical insult may have also led to an increased production of endogenous AVP from the supra-optic and paraventricular nucleus of the hypothalamus, which further precipitates hyponatraemia in the setting of partial AVP-D (10).

The treatment of multisystemic LCH involves the use of chemotherapy under the guidance of haematology. The chemotherapeutic agents that have been utilised in the treatment of multisystemic LCH include vinblastine, cladribine, and cytarabine. Allogenic haematopoietic stem cell transplant may be considered as salvage therapy in treatment-resistant LCH, and targeted therapy with BRAF inhibitors can be considered in multisystemic disease with CNS involvement (7).

In summary, we presented a case of a 48-year-old man with a concurrent diagnosis of papillary thyroid cancer and LCH with non-concordant BRAF mutation status. LCH poses a unique intraoperative challenge whereby it directly affects the tissue integrity, as seen in our case. Furthermore, endocrinopathies associated with LCH may further complicate perioperative care. Strict monitoring of fluid balance as well as serial monitoring of serum sodium are essential in all patients with AVP-D in the perioperative setting.

## Declaration of interest

The authors declare that there is no conflict of interest that could be perceived as prejudicing the impartiality of this case report.

## Funding

This case report received no specific funding.

## Patient consent

Written consent for publication of the article along with associated images was obtained directly from the patient on 2 February 2023.

## Author contribution statement

The co-authors of the case report were directly involved in providing clinical care to the patient. Each co-author made equal contribution to the writing of the case report.
